# Prevalence and management of anaemia in patients with non-myeloid cancer undergoing systemic therapy: a Spanish survey

**DOI:** 10.1007/s12094-012-0953-5

**Published:** 2012-12-19

**Authors:** J. L. Steegmann, J. M. Sánchez Torres, R. Colomer, Á. Vaz, J. López, I. Jalón, M. Provencio, A. González-Martín, M. Pérez

**Affiliations:** 1 Servicio de Hematología, Hematology Department, Instituto de Investigación Sanitaria (IIS-IP), Hospital Universitario de la Princesa, Diego de Leon, 62, 28006 Madrid, Spain; 2 Servicio de Oncología Médica, Hospital 12 de Octubre, Madrid, Spain; 3 Servicio Oncología Médica, M.D. Anderson Cancer Center, Madrid, Spain; 4 Departamento de Hematología, Hospital Ramón y Cajal, Madrid, Spain; 5 Departamento de Hematología, Clínica Ruber, Madrid, Spain; 6 Departamento de Oncología Médica, Hospital Puerta de Hierro, Madrid, Spain; 7 Servicio de Oncología, Hospital Universitario de la Princesa, Madrid, Spain; 8 Present Address: M.D. Anderson Cancer Center, Madrid, Spain

**Keywords:** Anaemia, Darbepoetin alfa, Chemotherapy, Solid tumours, Haematological malignancies, Transfusion

## Abstract

**Background:**

The present study aimed to provide updated data on anaemia prevalence and management in cancer patients undergoing systemic therapy in Spain.

**Methods:**

This was a multicenter, observational, cross-sectional study performed in 2008. Eligible patients were ≥18 years, with non-myeloid malignancies treated with systemic therapy [chemotherapy (CT), hormonal therapy or immunotherapy]. Anaemia was defined according to WHO as haemoglobin (Hb) < 12 g/dL.

**Results:**

The study included 214 patients with a median age of 63 years (range 20–91), 58 % women, 73 % with solid tumours, and 79 % with advanced disease. CT was used in 91 % of patients (26 % with platinum compounds), hormonal therapy in 8.5 %, and immunotherapy in 8.5 %. In our study, 48.1 % of patients [95 % confidence interval (CI) 45.2–58.6] showed anaemia (31 % symptomatic): 42.0 % mild (10 ≤ Hb ≤ 11.9 g/dL), 5.6 % moderate (8 ≤ Hb ≤ 9.9 g/dL), and 0.5 % severe (Hb <  8 g/dL). A higher prevalence was observed in patients treated with CT (51 vs. 20 %, *p* = 0.01), platinum-based CT (70 vs. 47 %, *p* = 0.01) or palliative CT (61 vs. 39 %, *p* = 0.003). Anaemia was also more frequent in patients with more than three lines of CT (83 %) and in the fourth or subsequent CT cycle (58 %). Management in the previous 4 weeks in patients with anaemia was: 62 % did not receive treatment (92 % mild), 24 % received erythropoiesis-stimulating agents (ESAs), 14 % received iron and 8.7 % received transfusion.

**Conclusions:**

In Spanish hospitals, about half of patients with non-myeloid malignancies undergoing systemic therapy fulfilled anaemia criteria (87 % mild). Approximately two-third of patients with anaemia do not receive specific treatment and ESA use is below current guidelines.

## Introduction

Most cancer patients suffer anaemia during the course of the disease or the treatment, in particular those patients receiving myelosuppressive chemotherapy [1]. Severe anaemia (<8 g/dL) can lead to the onset of cardiovascular and respiratory symptoms, such as tachycardia, hypotension and dyspnoea, although less severe anaemia can also produce a range of symptoms such as fatigue, exercise intolerance, decreased appetite and reduced general welfare [2]. In addition, anaemia has been shown to influence the evolution of disease, increasing the risk of progression and decreasing overall survival [3, 4].

Significant progress has been made in the prevention and management of chemotherapy-induced anaemia. Current therapeutic options include iron supplementation, administration of erythropoiesis-stimulating agents (ESAs) and blood transfusion. Several guidelines have been published with specific recommendations [5–7], like the 2007 European Organization for Research and Treatment of Cancer (EORTC) guidelines [6]. The European Cancer Anaemia Survey (ECAS) [8], the first major epidemiological study on anaemia conducted in 2001, revealed a high prevalence and incidence, and highlighted that many patients (some of them with haemoglobin [Hb] < 10 g/dL and/or symptomatic anaemia) received no specific treatment. Since then, some studies in particular countries like France [9], Germany [10] and Belgium [11], have provided updated and country-specific information on anaemia management and fulfilment of guidelines. In the Spanish context, there is no recent data available in this field. This study aims to provide accurate and relevant information on the prevalence and patterns of treatment of anaemia in Spanish cancer patients treated with systemic therapy with or without radiation therapy in the clinical practice.

## Patients and methods

This was an observational, multicenter, cross-sectional study in 21 Spanish hospitals. We consecutively included all patients who attended the clinic during a particular day of the week from May 19 to May 23, 2008 (to be chosen by each site according to their availability), and fulfilled selection criteria: age ≥18 years; patients diagnosed with non-myeloid tumour (except for myelodysplastic syndromes), undergoing treatment with systemic therapy (CT, hormone therapy or immunotherapy), with or without radiation therapy; patients who had received the last cycle of treatment within the previous 4 weeks; patients who had a blood test (including Hb value) performed on the date of the visit or within the past 72 h; non-hospitalized patients. The protocol was approved by an independent ethics committee, and all patients gave their written informed consent before enrolment.

Data on clinical characteristics, type of cancer, treatment for anaemia and Hb levels were collected for the baseline visit and for the past 4 weeks. We defined the degree of anaemia according to World Health Organization (WHO) as follows: mild anaemia, Hb between 11.9 and 10 g/dL; moderate anaemia, Hb between 9.9 and 8 g/dL; severe anaemia, Hb < 8 g/dL.

Statistical analysis was descriptive. The prevalence of anaemia in the entire study population and in specific subgroups (by type and stage of tumour, age category, sex, presence or absence of symptoms and type and characteristics of systemic therapy) was described. Treatments for anaemia received in the 4 weeks prior to inclusion and provided on that day were also described (no treatment, transfusion, iron supplements or vitamins, ESAs). We performed an exploratory analysis to analyze the relationship between patient characteristics or treatment and the presence of anaemia. For qualitative variables, the relationship was assessed using Chi Square tests (or Fisher’s exact test if it was applicable), and for quantitative variables, using Student’s *t* tests. Statistical analyses were performed with the SAS^®^ package-version 9.0 (SAS Institute, Cary, NC, USA).

## Results

### Study population

The study included 214 patients with a median age of 63 years (range 20–91), 58 % women and 73 % with solid tumours. The most common tumour types were breast (37.6 %), gastrointestinal (23.6 %) and lung cancer (19.7 %). Most patients (91 %) were receiving CT (of which 26 % received platinum compounds), 8.5 % hormone therapy, and 8.6 % immunotherapy (Table 1).

**Table 1:** Characteristics of the patients and treatment administered

	Non-myeloid malignancies *N* = 214		Non-myeloid malignancies *N* = 214
Age (years), median (range)	63 (20; 91)	Chemotherapy, *n* (%)	193 (91.0)
Women, *n* (%)	124 (57.9)	Adjuvant^c^	37 (20.7)
Reason for visit, *n* (%)		Advanced stage^c^	142 (79.3)
Chemotherapy	160 (74.8)	Line of advanced stage, *n* (%)^d^	
Monitoring visit	55 (25.7)	First	76 (53.9)
Radiotherapy	10 (4.7)	Second	37 (26.2)
Therapy (antiemetics…)	5 (2.3)	Third	16 (11.3)
Treatment of complications	1 (0.5)	More than third	12 (8.5)
RBC transfusion	1 (0.5)	Intention, *n* (%)^e^	
Other reasons	3 (1.4)	Curative	87 (46.8)
Time since diagnosis (years), median (range)	0.7 (0.1–20)	Palliative	99 (53.2)
Primary tumour, *n* (%)		Cycle number, median (range)	4 (1; 19)
Solid tumour	157 (73.4)	Type of therapy, *n* (%)^f^	
Breast	59 (37.6)	Monotherapy	69 (36.7)
Gastrointestinal	37 (23.6)	Polychemotherapy	119 (63.3)
Lung	31 (19.7)	Use of platinums, *n* (%)^g^	
Other	30 (19.1)	With platinum	40 (25.8)
Haematological tumour	57 (26.6)	Without platinum	115 (74.2)
Lymphoma	14 (24.6)	Radiotherapy, *n* (%)^h^	21 (10.0)
Myeloma	31 (54.4)	Hormonal therapy, *n* (%)^i^	18 (8.5)
Other	12 (21.0)	Immunotherapy, *n* (%)^j^	18 (8.6)
Metastasis (only solid tumours), *n* (%)^a^	98 (63.2)	Targeted therapy, *n* (%)^j^	20 (9.5)
Stage III–IV (only haematological tumours), *n* (%)^b^	27 (65.8)	Other systemic treatments, *n* (%)^j^	17 (8.1)

*ECOG* Eastern Cooperative Oncology Group, *RBC* red blood cells, *SD* standard deviation

^a^Valid *n* = 155, ^b^ valid *n* = 41, ^c^ valid *n* = 179, ^d^ valid *n* = 141, ^e^ valid *n* = 186, ^f ^valid *n* = 188, ^g^ valid *n* = 155, ^h^ valid *n* = 211, ^i^ valid *n* = 213, ^j^ valid *n* = 210

The main reason for the visit was CT administration (74.8 %), followed by routine follow-up visit (25.7 %), radiotherapy administration (4.7 %) and other reasons (5.2 %). The median time since diagnosis was 8.4 months (range 1.2–240).

### Prevalence of anaemia

**Fig. 1:**
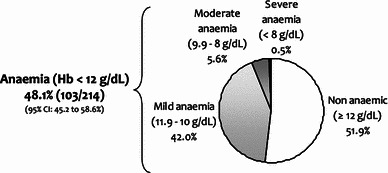
Prevalence of anaemia in cancer patients treated with systemic therapy

Overall, 48.1 % of patients (95 % CI 45.2–58.6) had anaemia (of whom 13 % had levels <10 g/dL and 31 % were symptomatic) (Fig. 1).

Median Hb level in the overall sample was 12.0 g/dL (range 7.4–16.1) (Table 2). The proportion of patients who reported symptoms of anaemia in the current visit was the same as in the previous 4 weeks. Only three patients with symptoms at the current visit did not have anaemia according to the laboratory test.

**Table 2:** Haematological parameters and symptoms of anaemia

Variable		
Haemoglobin (g/dL)	Valid *n*	214
	Mean (SD)	12.06 (1.60)
	Median (range)	12.0 (7.4; 16.1)
Leukocytes (×10^9^/L)	Valid *n*	214
	Mean (SD)	5.91 (2.97)
	Median (range)	5.0 (1.6; 17.7)
Platelets (×10^9^/L)	Valid *n*	214
	Mean (SD)	230.44 (115.49)
	Median (range)	213.0 (22.5; 927.0)
During the last 4 weeks, has the patient had symptoms related to anaemia?	Valid *n*	206 (100.0 %)
	Yes	34 (16.5 %)
	No	172 (83.5 %)
In the current visit, has the patient symptoms related to anaemia?	Valid *n*	208 (100.0 %)
	Yes	35 (16.8 %)
	No	173 (83.2 %)

*SD* standard deviation

### Factors related to anaemia

**Fig. 2:**
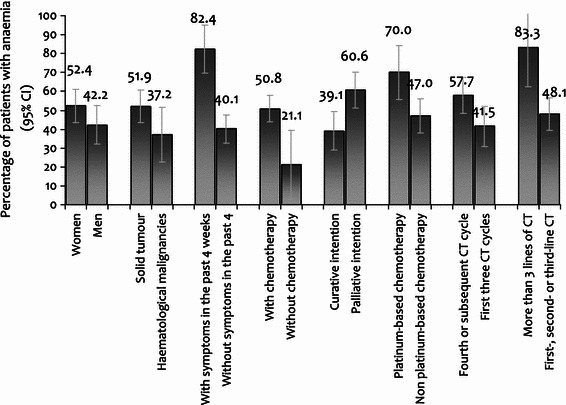
Prevalence of anaemia in different subgroups according to patient characteristics, type of tumour, symptoms related to anaemia and systemic therapy

There was a trend towards a higher prevalence of anaemia in women (52 vs. 42 % in men, *p* = 0.140) and in patients with solid tumours (52 vs. 37 % in patients with haematological malignancies, *p* = 0.094) (Fig. 2). No association between anaemia and age, stage or tumour type was observed (data not shown). A higher prevalence was found in patients with symptoms of anaemia in the last 4 weeks (82 vs. 40 % in patients without symptoms, *p* < 0.0001), and in patients treated with CT (51 vs. 21 % in patients treated with hormonal therapy or immunotherapy, *p* = 0.013), platinum-based CT (70 vs. 47 % in patients with non-platinum based CT, *p* = 0.013) or palliative CT (61 vs. 39 % in patients with curative CT, *p* = 0.003) (Fig. 2).

There were no differences in the prevalence of anaemia between patients with CT receiving monotherapy or combination therapy (52 vs. 49 %, *p* = 0.457). The administration of concomitant radiotherapy was not related to anaemia either (52 vs. 48 %, *p* = 0.944).

The proportion of patients with anaemia was significantly higher in patients with more than three lines of CT (83 vs. 46, 54 and 44 % in patients undergoing first-, second- and third-line of CT, respectively, *p* = 0.006) and in patients in the fourth or subsequent CT cycles, compared with patients in the first three cycles (58 vs. 41 %, *p* = 0.028).

No significant differences were found in mean leukocyte or platelet count between patients with and without anaemia (data not shown).

### Anaemia management

**Fig. 3:**
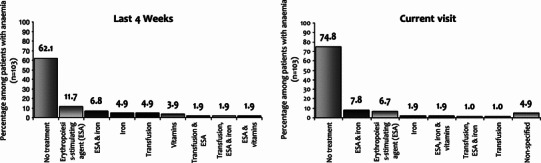
Therapeutic management of anaemia (previous 4 weeks and current visit) in patients with Hb < 12 g/dL at study inclusion (*n* = 103)

In patients with anaemia (*n* = 103), the management in the previous 4 weeks was as follows: 62.1 % received no specific treatment (of whom 91.9 % had mild anaemia), 24.0 % received ESA, 13.6 % received iron supplementation and 8.7 % received transfusion (Fig. 3). The use of transfusion in the previous 4 weeks was higher in patients with moderate or severe anaemia, compared to patients with mild anaemia (23.1 vs. 5.6 %, *p* < 0.0001). Thereby, 37.5 % of patients who received transfusion during the previous 4 weeks (3 out of 8) still had moderate or severe anaemia.

In the current visit, the percentage of patients without any treatment for anaemia increased to 74.8 % (of whom 94.9 % had mild anaemia). Sixty-nine percent of the patients with moderate or severe anaemia received treatment, compared with only 18 % of patients with mild anaemia (*p* < 0.0001). Only two patients were prescribed a transfusion at the study visit (one received only transfusion and the other one received transfusion, iron and ESA), both having moderate or severe anaemia.

Among the 25 patients who received any anaemia treatment at the study visit, 18 patients received ESA’s (17.5 % of anaemic subjects). Darbepoetin alfa was the most commonly used agent (13 patients, four patients received epoetin beta, and 1 patient received epoetin alfa). The use of ESA’s was more frequent in patients with moderate or severe anaemia (46 vs. 13 % in patients with mild anaemia, *p* = 0.012). Among patients prescribed ESA’s, the mean Hb in patients with mild or moderate-severe anaemia was 10.5 and 8.2 g/dL, respectively. Iron supplementation was used in 13 patients (12.6 % of anaemic subjects).

## Discussion

We present information on the prevalence and management of anaemia in an unselected cohort of cancer patients undergoing systemic therapy in the Spanish setting. We found a prevalence of anaemia (defined as Hb < 12 g/dL) of approximately 50 %, which is similar to that found in other contemporary studies from European countries [9–11].

In the ECAS, conducted in 2001, the prevalence of anaemia at enrolment was lower (39 %), but the proportion of anaemic subjects not receiving treatment was comparable to ours (more than 60 %) [8]. Recent data from France suggest a better management of anaemia in this country, with ESA administration in approximately two-third of anaemic patients, and only 17 % non-treated [9]. In the Belgian study with the same design as the present one, the prevalence of anaemia was comparable (56 %), but a higher relative proportion of moderate versus mild anaemia was found (threefold to that of the Spanish cohort) [11]. They also observed more anaemia in patients with haematological malignancies (73 %) compared to solid tumours, which disagrees with our results. The fact that they did not exclude patients with myelodysplastic syndromes (at high risk of anaemia) can partially explain this finding, but probably there are other differences involved [for example, more use of platinum-based regimens (37 vs. 26 % in our cohort), or more proportion of advanced disease]. Regarding anaemia management, there were also important disparities with our results: a higher proportion of treated patients (almost 50 %) and more transfusions. Iron use was similar [11].

We observed a strong association between CT administration and anaemia, as previously reported in the ECAS [12]. Among CT-related factors, more than three lines of treatment or more than three cycles were associated to a higher risk of anaemia. Similarly, during ECAS, the prevalence of anaemia increased progressively from cycle 1 to cycle 6 [13]. The observed association with palliative CT probably reflects the degree of severity of the disease and its impact on erythropoiesis. Interestingly, the prevalence of anaemia was not different in patients receiving mono-CT as opposed to poly-CT, but it was higher in patients receiving platinum-based regimens (70 vs. 47 %). We did not find any significant influence of radiotherapy administration. A model developed by Barret et al. [14] to predict anaemia development in cancer patients initiating CT included the following variables as the most important factors: low initial Hb (≤12.9 g/dL in females, and ≤13.4 g/dL in males); cancer type other than gastrointestinal/colorectal cancer; treatment with platinum CT, and female gender. We did not analyze the prevalence by tumour subtype, due to sample size limitation, but we confirm a strong association with platinum-based CT.

Since ECAS, several guidelines have provided recommendations for ESA use in cancer patients. The EORTC [6] guidelines recommend to treat all patients with Hb levels below 9 g/dL (who were only a small percentage of our sample), and all symptomatic patients with levels between 9 and 11 g/dL. The Anaemia Cancer Treatment (A.C.T.) study was a retrospective, extensive review of anaemic patients with cancer treated with ESAs investigating patterns of use according to EORTC guidelines [15]. It concluded that, in 2007, most European patients were treated per guidelines, except for low iron supplementation rates [16]. A prospective German study in cancer patients with anaemia observed that physicians preferred ESA as first-line treatment in patients with solid tumours, but in patients with lymphoproliferative malignancies, transfusions or correction of underlying disorders was preferred. They also reported underuse of intravenous iron therapy [10]. In our cohort, around one-third of anaemic subjects were symptomatic, which means that at least the same amount of individuals would benefit from receiving ESAs. Since only 17 % of patients were being treated with erythropoietic agents, we can conclude that the EORTC guidelines were still not fully implemented during 2008 in the clinical practice of Spanish Oncology or Oncohematology services. Current guidelines of the Spanish Society of Medical Oncology (SEOM) about the use of ESAs in cancer patients [17] were published in 2009, after the study completion. They established that, since ESA treatment increases the Hb level and decreases the red blood cell transfusion requirements, ESAs should be used within the approved indications in patients undergoing chemotherapy treatment, beginning at an Hb level below 11 g/dL and maintaining it around 12 g/dL, with iron supplements if necessary. Thus, our data also shows that management of anaemia in Spain needs to be updated according to local guidelines.

Despite the concerns about use of ESAs, all meta-analyses considering only clinical trials performed under the approved indication (patients receiving chemotherapy) support their safety and clinical benefit in the treatment of chemotherapy-induced anaemia [18–23], including a reduction in transfusion needs both in solid tumours and in haematological malignancies [24, 25]. In the larger meta-analysis conducted until now, the use of ESAs was neutral with regard to survival and disease progression [26]. In this meta-analysis, it was also demonstrated that the use of ESAs is associated with an increased risk of venous thromboembolism (odds ratio = 1.4) [26]. Regarding the increase in the risk for thromboembolic events [23, 27], it must be taken into account specially in patients with cardiovascular disease, in whom the ESA dose must be adjusted according to patient characteristics to avoid an excessive change in plasma volume and maintaining a consistent Hb level [28].

The main limitations of our study are the limited sample size, that does not allow to explore differences in the prevalence and management of anaemia between tumour subtypes, for example, and the cross-sectional design, which does not allow for causal inferences.

## Conclusion

In Spanish hospitals, about half of patients with non-myeloid malignancies undergoing systemic therapy suffer from anaemia. Chemotherapy administration, especially if it contains platinum compounds or represents an advanced-line therapy, is associated with a high risk of anaemia. Our results show that approximately two-third of patients with anaemia do not receive specific treatment and that ESAs are underused according to current EORTC and local SEOM guidelines. Since the treatment of anaemia with ESAs (and adequate iron supplementation) can significantly improve patients’ quality of life and may also improve the clinical outcome, it is advisable to review and optimize anaemia management in the clinical practice of Spanish Oncology and Hematology services.

## Appendix

The following investigators are members of the ANAEMIA DAY Study Group and also participated in this study: Co-authors. This study has been conducted by the above-signed authors and the ANAEMIA DAY study group: Dra. Carmen Crespo; Dr. Javier López; Dr. J. Sánchez Torres; Dr. Ramón Colomer; Dra. Ángeles Vaz; Dr. Ignacio Jalón; Dr. Mariano Provencio; Dra. Mar Pérez; Dr. Jaime Pérez; Dr. J. L. Steegmann; Dr. J. A. Arranz y Dr. Luís Cabezón; Dr. Carlos Grande; Dra. Ana I. Ballesteros; Dr. Javier Puente; Dra. Concha López; Dra. Anabelle Chinea y Dr. Valentín Gutiérrez; Dr. Pedro Salinas y Dr. Pérez Carrión; Dra. Raquel de Paz y Dra. Ana López; Dr. Ignacio García E; Dra. Pilar Samper; Dr. J. A. García Saénz.
